# Conductivity-limiting bipolar thermal conductivity in semiconductors

**DOI:** 10.1038/srep10136

**Published:** 2015-05-13

**Authors:** Shanyu Wang, Jiong Yang, Trevor Toll, Jihui Yang, Wenqing Zhang, Xinfeng Tang

**Affiliations:** 1 Materials Science and Engineering Department, University of Washington, Seattle, WA 98195-2120, USA; 2 State Key Laboratory of High Performance Ceramics and Superfine Microstructure, Shanghai Institute of Ceramics, Chinese Academy of Sciences, Shanghai 200050, China; 3 State Key Laboratory of Advanced Technology for Materials Synthesis and Processing, Wuhan University of Technology, Wuhan 430070, China

## Abstract

Intriguing experimental results raised the question about the fundamental mechanisms governing the electron-hole coupling induced bipolar thermal conduction in semiconductors. Our combined theoretical analysis and experimental measurements show that in semiconductors bipolar thermal transport is in general a “conductivity-limiting” phenomenon, and it is thus controlled by the carrier mobility ratio and by the minority carrier partial electrical conductivity for the intrinsic and extrinsic cases, respectively. Our numerical method quantifies the role of electronic band structure and carrier scattering mechanisms. We have successfully demonstrated bipolar thermal conductivity reduction in doped semiconductors via electronic band structure modulation and/or preferential minority carrier scatterings. We expect this study to be beneficial to the current interests in optimizing thermoelectric properties of narrow gap semiconductors.

Thermal conduction in solids is one of the most fundamental physical processes. It reveals the nature of lattice dynamics as well as phonon scattering mechanisms. Thermal conductivity of solids also influences many technologically important topics including thermal insulation and management of energy storage and conversion systems, microelectronics, data storage devices; efficiency of thermoelectric materials; and stability of sensors and actuators. For semiconductors the low temperature thermal conductivity is not substantially distinct from those of insulators; at elevated temperatures, however, it becomes interesting and yet intriguing due to the vital roles of charge carriers and their interactions. A signature of electron-hole coupling in semiconductors is the bipolar thermal conduction at elevated temperatures, when the calculated lattice thermal conductivity (*κ*-*Lσ*Τ, where *κ* is the total thermal conductivity, *L* the Lorenz number, *σ* the electrical conductivity, and *T* the absolute temperature) is significantly higher than the *T*^−*1*^ temperature dependence expected for phonon-phonon interaction dominated thermal conductivity^1^,^2^,^3^,^4^,^5^,^6^,^7^. Similar effect has also been found in semimetals^8^,^9^,^10^. For intrinsic semiconductors, it is well recognized that the mobility ratio between electrons and holes (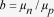) determines the bipolar thermal conductivity (*κ*_*b*_), which maximizes when *b* = 1^11^,^12^. Consequently, *κ*_*b*_ is insignificant for InSb, primarily due to its very large mobility ratio (*b* > 100)^13^. In the case of heavily doped semiconductors, the mobility ratio however is no longer a valid guide for understanding or predicting *κ*_*b*_, due to the substantially different majority and minority carrier concentrations. For example, recent experiments showed significant *κ*_*b*_ in *p*-ty*p*e heavily-doped skutterudites despite of the mobility ratio between two carriers being greater than 10 (hole mobility ~1–5 cm^2^/V-s with a concentration of ~10^21^ cm^−3^ and electron mobility ~30–50 cm^2^/V-s with a concentration of ~10^18^–10^19^ cm^−3^ at 800 K, according to our numerical analyses which are presented below)^14^,^15^,^16^,^17^,^18^, while the *n-type* skutterudites do not show appreciable *κ*_*b*_, consistent with the rather small *b* value (~1/50)^19^,^20^,^21^,^22^,^23^,^24^,^25^,^26^,^27^,^28^,^29^. Similar observations have been reported for many other semiconductors^30^,^31^,^32^,^33^,^34^,^35^,^36^,^37^,^38^,^39^. These intriguing results necessitate comprehensive understanding of *κ*_*b*_ in semiconductors. A recent report attempted to model *κ*_*b*_ in doped Bi_2_(Te_0.85_Se_0.15_)_3_ crystals but was unable to capture the specific roles of electronic band structure and carrier scattering mechanisms on *κ*_*b*_^35^.

In this study we report a combined experimental and computational effort that focused on unraveling the general behavior of *κ*_*b*_ in semiconductors. A numerical method for modeling the temperature dependence of *κ*_*b*_ for intrinsic as well as extrinsic (heavily doped) semiconductors encompassing a wide range of band gap and electronic band structure has been developed. We find that *κ*_*b*_ in semiconductors is in general “conductivity-limiting”. In analogous to the bipolar ionic conduction and multiple-step diffusion processes, in which the overall kinetics are determined (limited) by the lower rate species or processes, the bipolar thermal conduction is limited by the charge carrier with lower partial electrical conductivity^40^,^41^. Therefore, it is determined by the minority carrier partial electrical conductivity and by the mobility ratio (“mobility-limiting”) in extrinsic and intrinsic semiconductors, respectively. In order to validate these findings, we experimentally demonstrated *κ*_*b*_ reduction based on electronic band structure modulation and preferential minority carrier scattering. These results largely broaden our understanding of thermal conduction in semiconductors as well as offer insights for optimizing thermoelectric properties of narrow gap semiconductors.

## Results and Discussion

Bipolar thermal conductivity in semiconductors can be expressed as^1^,^2^,^3^,^4^


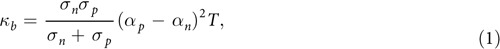


where *σ*_*i*_ and α_*i*_ (subscript *i* = *n*, *p*) are the partial electrical conductivity and Seebeck coefficient for electrons and holes, respectively. For a single parabolic band, the Seebeck coefficient of each carrier can be written as^42^


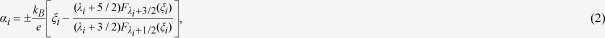


where *k*_*B*_ is the Boltzmann constant, *e* the free electron charge, *ξ* the reduce Fermi energy, *λ* the carrier scattering parameter, *F*_*x*_ the Fermi integral of the order of *x*. Therefore 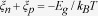, where *E*_*g*_ is the band gap^4^. For acoustic phonon scattering (*λ*  = −1/2), the term 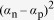 can be written as 
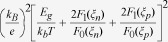
 which is associated with the total energy carried by electron-hole pairs (band gap energy and kinetic energies). The electrical conductivity of each carrier is


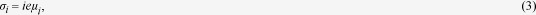


where *i* *=* *n*, *p* designates the carrier concentrations of electron and hole, respectively.

### “Conductivity-Limiting” Bipolar Thermal Conductivity

To elucidate the bipolar thermal conduction behavior in semiconductors, we may rearrange Eq. (1) into (assuming acoustic phonon scattering *λ* = −1/2, which is valid for most thermoelectric materials)


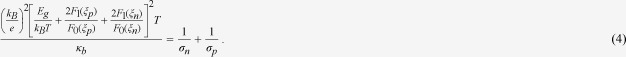


**Figure 1:**
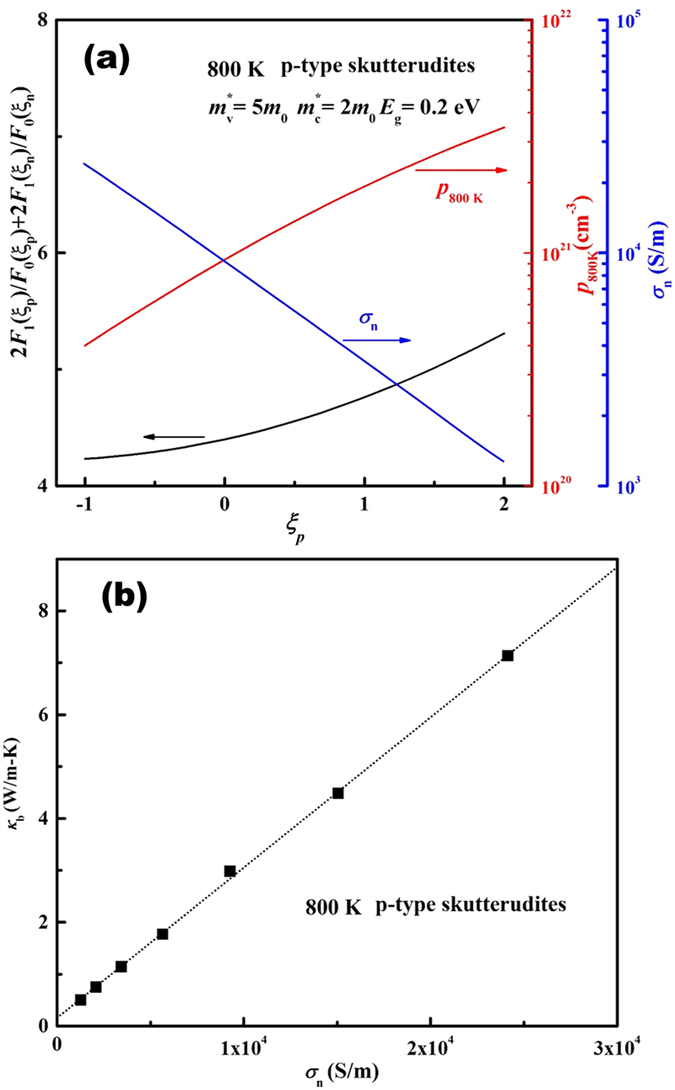
(**a**) Numerically calculated total reduced kinetic energy 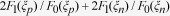 for holes and electrons, hole (majority carrier) concentration, and electron (minority carrier) partial electrical conductivity as a function of the reduced Fermi level (*ξ*_p_); (**b**) calculated *κ*_b_ as a function of minority carrier partial electrical conductivity *σ*_n_. The dashed line is a guide for eye. The calculations were carried out for *p*-*type* skutterudites (R_x_Fe_3_NiSb_12_) with *E*_g_ = 0.2 eV, *m*_p_^*^ = 5*m*_0_, *m*_n_^*^ = 2*m*_0_, at 800 K.

For a given material at a fixed *T*, the variation of 
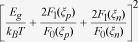
 as a function of *ξ*_*p*_ or *ξ*_*n*_ is rather negligible, while the carrier concentrations and the partial electrical conductivity *σ*_*i*_ (right side of Eq. (4)) could change by several orders of magnitude because of the activation behavior of the charge carriers. Here 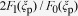 and 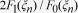 are the reduced kinetic energies of holes (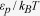) and electrons (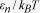), respectively, which only slightly change their numerical values when varying the Fermi level^43^. To verify these analyses, numerical data for *p*-type skutterudites (*R*_x_Fe_3_NiSb_12_) with *E*_*g*_ = 0.2 eV, *m*_p_^*^ = 5 *m*_*0*_, *m*_n_^*^ = 2 *m*_*0*_, at 800 K are plotted in fig. 1, where *m*_p_^*^, *m*_n_^*^, and *m*_*0*_ are the effective mass of holes, effective mass of electrons, and free electron mass, respectively. The details of the calculations will be discussed below. As shown in fig. 1(a), with increasing *ξ*_*p*_ from –1 (weakly-degenerate) to 2 (degenerate), 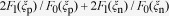 only increases from 4.2 to 5.3, ~25% increases; whereas *p* increases by a factor of ~10 and the minority carrier partial conductivity *σ*_*n*_ decreases by a factor of ~20. These suggest that for semiconductors in general, Eq. (1) or (4) can be approximated as 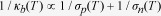, therefore *κ*_*b*_ in semiconductors is actually “conductivity-limiting”, analogous to the rate-limiting phenomena in kinetic diffusion processes^41^. For intrinsic semiconductors, since *n* = *p*, Eq. (4) can be further approximated to be 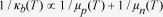, consistent with the large body of literature already developed. In the case of extrinsic semiconductors (*n* ≫*p* or *p* ≫*n*) *κ*_*b*_ is primarily determined by the partial electrical conductivity of the minority carriers, not by the mobility ratio. A linear dependence of *κ*_*b*_ vs. *σ*_*n*_ at 800 K for *p*-type doped skutterudites, as shown in fig. 1(b), further substantiates our proposed “conductivity-limiting” concept for bipolar thermal conduction in semiconductors.

### Numerical Modeling

**Figure 2:**
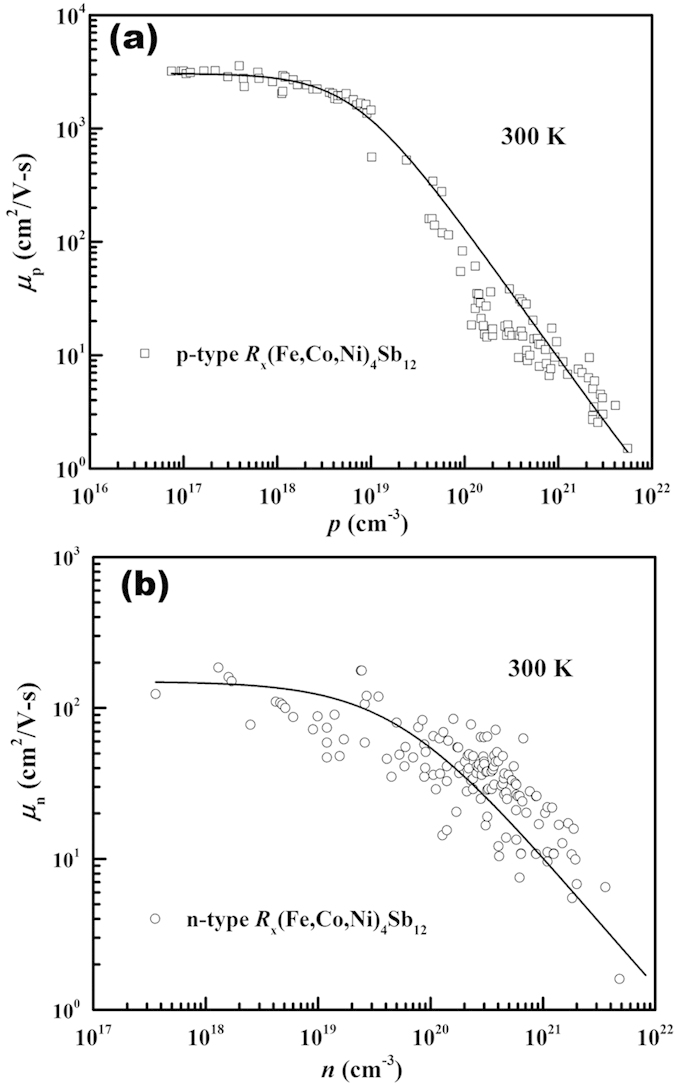
Room temperature carrier mobility as a function of carrier concentration for (**a**) *p-type* and (**b**) *n-type R*_x_(Fe,Co,Ni)_4_Sb_12_ skutterudites. The solid lines are least squares fits to the data using Eq. (5). Data used here are taken from Refs. 14, 15, 16, 17, 18, 19, 20, 21, 22, 23, 24, 25, 26, 27 and 47, 48, 49, 50, 51, 52, 53, 54, 55, 56, 57, 58, 59.

Data presented in fig. 1 were calculated by our numerical method for modeling the temperature dependence of *κ*_*b*_ in semiconductors. Our numerical method aimed at discerning the underlying physics that controls *κ*_*b*_, including the electronic band structure features and carrier scattering mechanisms. We use the experimental carrier concentration values as those of the majority carriers. Based on the majority carrier concentration and Seebeck coefficient at room temperature, and the maximum Seebeck coefficient value at elevated temperatures, we can determine the Fermi level, the majority carrier effective mass and *E*_*g*_^44^,^45^. The minority carrier effective mass is used as an adjustable parameter. The majority and minority carrier concentrations and their temperature dependences are calculated based on semiconductor statistics^46^. In order to obtain the *T* dependence of mobility, we first modeled its carrier concentration dependence at room temperature. We then assumed that the carriers are predominantly scattered by the acoustic phonons, therefore 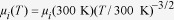 and 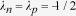. For example, the room temperature carrier mobility of *n-type* and *p*-type 3*d* transition metal-based skutterudite antimonides *R*_*x*_(Fe,Co,Ni)_4_Sb_12_ as a function of carrier concentration is shown in fig. 2, where *R* represents fillers and *x* the filling fraction. The data were taken from the literatures^14^,^15^,^16^,^17^,^18^,^19^,^20^,^21^,^22^,^23^,^24^,^25^,^26^,^27^,^47^,^48^,^49^,^50^,^51^,^52^,^53^,^54^,^55^,^56^,^57^,^58^,^59^ and were well represented by an empirical expression (the solid lines in fig. 2)^60^


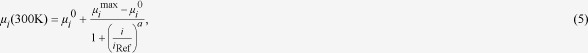


where *i*_*Ref*_ is the reference carrier concentration, approximately where degeneracy sets in, *a* is a fitting parameter, and 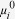 and 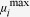 are the minimum and maximum possible mobility, respectively. In general, the carrier concentration dependence of mobility for all semiconductors studied in this work can be well accounted by this phenomenological formula and the fitting parameters are summarized in the Table S1 (Supporting Information, SI).

**Figure 3:**
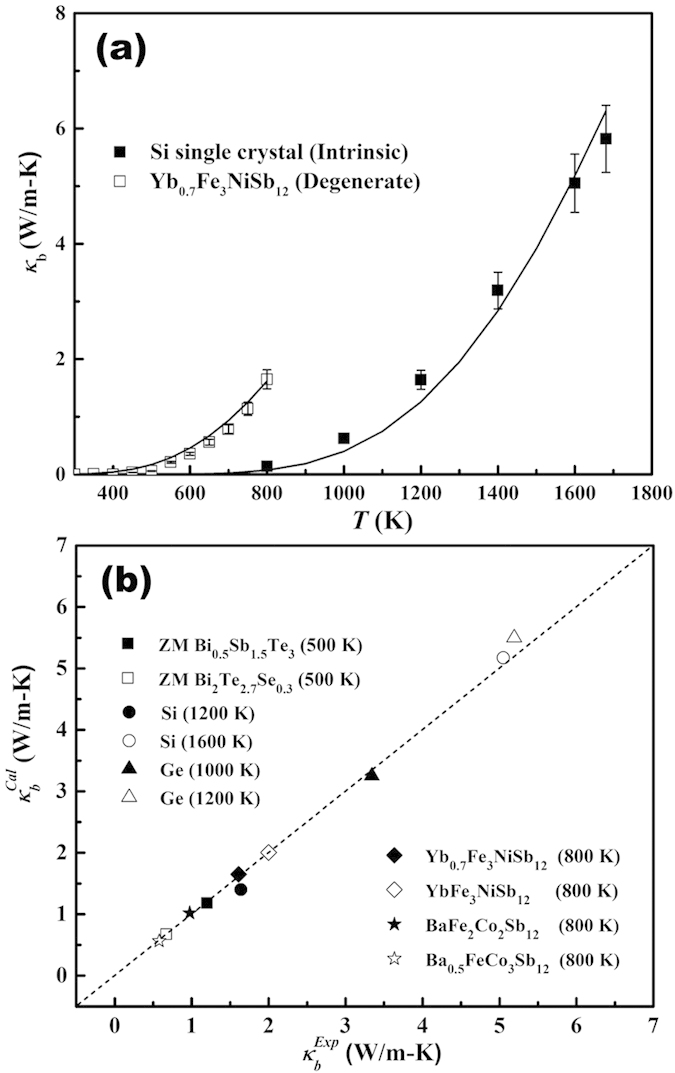
(**a**) Experimental (symbols) and fitted (solid lines) bipolar thermal conductivity of intrinsic Si single crystal and degenerate Yb_0.7_Fe_3_NiSb_12_ vs. *T*. (**b**) Experimental (*κ*_*b*_^*Exp*^) and calculated (*κ*_*b*_^*Cal*^) bipolar thermal conductivity for intrinsic Si and Ge single crystals, and degenerate Bi_2_Te_3_-based zone melted (ZM) compounds and *p-type* skutterudites at various temperatures. The dashed line represents *κ*_*b*_^*Cal*^ *=* *κ*_*b*_^*Exp*^.

Based on Eqs. (1)–(3) and (5) and the aforementioned method, we were able to numerically fit the temperature dependence of *κ*_*b*_ in intrinsic and extrinsic semiconductors with a large variation of band gap, the Fermi level, and effective mass values. Figure 3(a) shows the excellent agreement between the experimental data (symbols) for intrinsic Si single crystal^7^ and degenerate polycrystalline skutterudite Yb_0.7_Fe_3_NiSb_12_ in a wide temperature range. Figure 3(b) shows the calculated (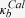) and experimental (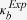) values of *κ*_*b*_ for a variety of materials at various temperatures, including (Bi,Sb)_2_(Te,Se)_3_^37^,^61^, skutterudites, Si, and Ge^6^,^7^. The dashed line in fig. 3(b) represents 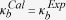. These results suggest that our method well accounts for the temperature dependence of *κ*_*b*_ in semiconductors (all relevant parameters used in our calculations are summarized in Table S2, SI). Since *κ*_*b*_ is determined by the minority carrier partial electrical conductivity in doped semiconductors, the minority carrier effective mass and its mobility, as well as *E*_*g*_ will have strong influence. The extent to which these parameters affect *κ*_*b*_ is illustrated in Fig. S1 (SI). This also suggests that *κ*_*b*_ modification can be achieved by manipulating these parameters.

### Bipolar Thermal Conductivity Reduction

**Figure 4:**
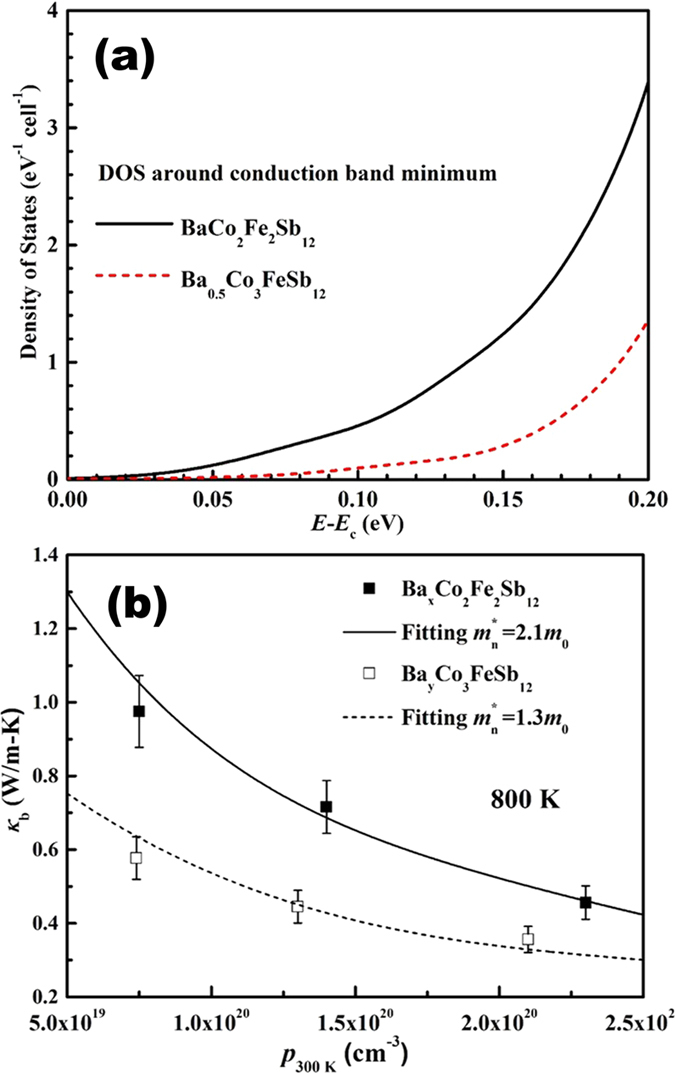
(**a**) The density of states around the CBM for BaCo_2_Fe_2_Sb_12_ and BaCo_3_FeSb_12_. (**b**) Bipolar thermal conductivity at 800 K as a function of hole (majority carrier) concentration for Ba_x_Co_2_Fe_2_Sb_12_ and Ba_y_Co_3_FeSb_12_. The lines in (**b**) are fits to the data using different minority carrier effective mass values.

In order to examine the validity of the minority carrier dominated bipolar thermal conduction in heavily doped semiconductors, and to utilize the concept of modifying *κ*_*b*_ presented, we investigated ways of *κ*_*b*_ reduction motivated by the recent quest for high efficiency thermoelectric materials that necessitate low thermal conductivity^62^,^63^,^64^,^65^. It is well known that in filled skutterudites^66^, the triple degenerate conduction band minimum (CBM) is primarily composed of *d*-orbitals from the transition metals (TMs), with some contribution from Sb *p*-states (*p*-*d* hybridization). Thus the density of states (DOS) at the CBM can be effectively adjusted by varying the TMs. Our first principles calculations reveal that in the *p*-type Ba-filled skutterudites, DOS at the CBM decreases significantly with decreasing Fe/Co ratio on the TM sites from 2:2 to 1:3, as shown in fig. 4(a), mainly due to the higher energy and thus more contribution of 3*d* orbitals of Fe as compared with those of Co. The distinct DOS of minority carrier band further suggests that *κ*_*b*_ for *p*-type Ba_0.5_Co_3_FeSb_12_ should be smaller than BaCo_2_Fe_2_Sb_12_ due to the minority carrier partial conductivity reduction. Data for 800 K *κ*_*b*_ vs. the majority carrier (hole) concentration for a series of Ba_x_Co_3_FeSb_12_ and Ba_y_Co_2_Fe_2_Sb_12_ samples are plotted in fig. 4(b), and the lines represent fitting to the data using the minority carrier effective masses 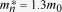 and 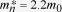, respectively. This electronic band modulation induced *κ*_*b*_ reduction substantiates the dominant role of the minority carriers. Because of the commonly triple-degenerate and 3*d-*orbital-dominated nature of the CBM, the minority carrier effective masses of the *p-type* skutterudites are usually much higher than those of the *n-type*, in which the minority carrier band is mainly composed of single-degenerate Sb *p*-orbital-featured light bands^67^. Therefore, the predominant underlying reason for large differences in *κ*_*b*_ between the *n*- and *p*-type skutterudites is actually due to the effective mass differences between the corresponding conduction and valence (minority) bands.

**Figure 5:**
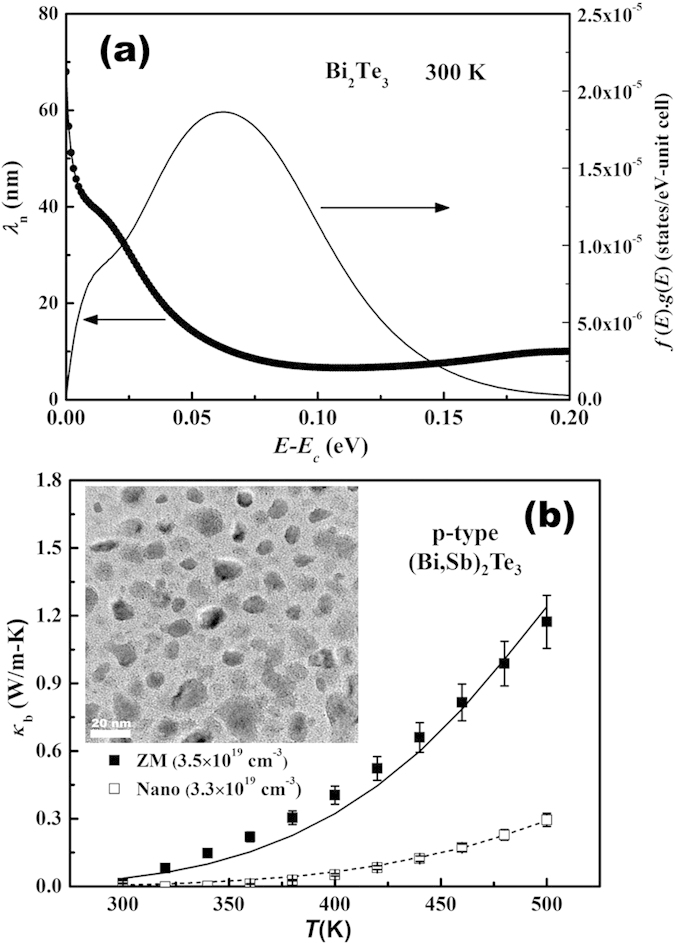
(**a**) The calculated electron wavelength, and the product of the Fermi-Dirac distribution function and electronic density of states *f(E) g(E)* vs. energy with the zero point corresponding to the conduction band minimum (*E*_*c*_). (**b**) The experimental and modeled bipolar thermal conductivity vs. temperature, for *p-type* zone melted (ZM) and nanostructured (MS-SPS) Bi_0.5_Sb_1.5_Te_3_. The inset is a TEM picture of the MS-SPS bulk sample which shows 10–50 nm nanoprecipitates. (The room temperature minority carrier mobilities of ZM and Nano samples are *μ*_*n*_ = 4095 and 1115 cm^2^/V-s, respectively).

Our second example of *κ*_*b*_ reduction takes the advantage of preferential scattering of the minority carriers. Normally in heavily doped semiconductors, the minority carriers are non-degenerate. Given the electronic band structure of a material and the Fermi level (determined by the majority carrier concentration), one can calculate the range of minority carrier wavelength^46^. For example, the electron wavelength in a heavily-doped *p-type* Bi_2_Te_3_ (*p* = 3.5 × 10^19^ cm^−3^) is approximately between 10 nm and 50 nm, as shown in fig. 5(a). We compare *κ*_*b*_ of *p*-type zone melted (ZM) and nanostructured (Nano) Bi_0.5_Sb_1.5_Te_3_ prepared by the melt spinning combined with subsequent spark plasma sintering (MS-SPS) technique^61^. Figure 5(b) shows, at comparable majority carrier concentrations between the ZM and Nano samples, a significant *κ*_*b*_ reduction is achieved when nanoprecipitates are introduced into the sample. The minority carrier partial electrical conductivity is determined by *E*_*g*_, minority effective mass and mobility. The estimated small *E*_*g*_ variation between ZM and Nano is only responsible for 20% of the *κ*_*b*_ reduction. For a large system like nanostructured Bi_0.5_Sb_1.5_Te_3_, a full electronic band structure calculation is computationally unfeasible. It is difficult to directly determine *m*_n_^*^ (minority carrier) at the CBM. The estimated *m*_n_^*^ values of *n-type* doped ZM and Nano Bi_2_Te_2.7_Se_0.3_ are 1.0 *m*_0_ and 1.1 *m*_0_, respectively^38^,^68^. If we assume comparable *m*_n_^*^ at CBM between the ZM and Nano samples, the major part of *κ*_*b*_ reduction between the *p-type* ZM and Nano Bi_0.5_Sb_1.5_Te_3_ with comparable majority hole concentrations could be attributed to the reduction of minority carrier mobility (*μ*_*n*_) corroborated by our *κ*_*b*_ fittings, where *μ*_*n*_ = 4095 cm^2^/V-s for the ZM and 1115 cm^2^/V-s for the Nano. The TEM image (inset of fig. 5(b)) shows that the sizes of nanoprecipitates closely match those of the minority electron wavelengths. Given the majority hole wavelength is estimated to be ~2 nm, we postulate a strong preferential minority carrier scattering by the nanoprecipitates in the Nano Bi_0.5_Sb_1.5_Te_3_. Similar *κ*_*b*_ reduction can also be observed in nanostructured *n-type* Bi_2_(Te,Se)_3_ compounds^38^,^69^,^70^. Extensive recent studies have established the role of nanostructure on lattice thermal conductivity reduction^63^,^65^, we propose an “preferential minority carrier scatterings” for *κ*_*b*_ reduction, which is partially responsible for the thermoelectric performance gains reported, especially at elevated temperatures^61^,^71^. Recent theoretical work has also demonstrated that similar *κ*_*b*_ reduction via heterostructure barriers scattering is possible^72^. Finally we caution that nanostructure induced band structure modulation reported in AgPb_m_SbTe_2-m_ might be possible for Bi_0.5_Sb_1.5_Te_3_^73^, which could be responsible for part of the *κ*_*b*_ reduction.

## Summary

To conclude, our combined theoretical analysis and experimental measurements have established that in semiconductors bipolar thermal transport is in general a “conductivity-limiting” phenomenon, which is controlled by the carrier mobility ratio and the minority carrier partial electrical conductivity for the intrinsic and extrinsic cases, respectively. The numerical method we developed quantifies the role of electronic band structure and carrier scattering mechanisms. We have also demonstrated feasible strategies for manipulating the bipolar thermal conductivity in doped semiconductors via electronic band structure modulation and/or preferential minority carrier scatterings. We expect our study to be beneficial to the current interests in optimizing thermoelectric properties of narrow gap semiconductors.

## Methods

Samples in this study were synthesized by a combination of induction melting and long-term high-temperature annealing, by zone melting, or by MS-SPS, and the details of which were documented elsewhere^19^,^61^. High-resolution transmission electron microscopy (TEM) images were collected using a JEM-2100F TEM. Electrical conductivity (*σ*) and Seebeck coefficient (α) were simultaneously measured by an Ulvac ZEM-3 under a low-pressure helium atmosphere. Thermal conductivity was calculated from the measured thermal diffusivity (*D*), specific heat (*C*_p_), and density (*d*) using the relationship *κ* = *DC*_p_*d*. Thermal diffusivity *D* was tested by laser flash diffusivity method using a Netzsch LFA-457 system, and *C*_p_ was measured by a Netzsch DSC 404F1 using sapphire as the reference. The accuracy of the *κ* measurements is estimated to be ~10% and the precision <5%. *κ*_*b*_ were extrapolated from *κ*_*b*_ + *κ*_*L*_ = *κ-LσT* by assuming lattice thermal conductivity *κ*_*L*_ is inversely proportional to *T*. Hall measurements were performed on a Janis cryostat equipped with a 9 Tesla superconducting magnet. The carrier concentration of electron (*n*) or hole (*p*) and the corresponding Hall mobility *μ*_*n*_ or *μ*_*p*_ (subscript *n* represents the electron and *p* the hole) were estimated from the measured Hall coefficient (*R*_*H*_) and electrical conductivity by the relation 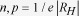 and 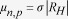, respectively.

The first-principles electronic band structure calculations were performed with the generalized gradient approximation functional of Perdew, Burke, and Ernzerhof^74^, with projected augmented wave method^75^,^76^, as implemented in Vienna *ab initio* simulation package (VASP)^77^. The computational techniques are similar to those published previously^66^,^78^. The de Broglie wavelengths (*λ*) is defined as, *λ* = *h*/m^*^v, where *h*, m^*^, and *v* are the Planck constant, carrier effective mass, and drift velocity, respectively. m^*^, v and *λ* of degenerate majority carriers are almost energy independent (*k*_B_*T* within the Fermi level), while for non-degenerate minority carriers these values are energy dependent, which are derived from band structure. The detailed calculation method is shown in Supporting Information, and the calculated density of state, *m*_n_^*^ and *v*_*n*_ of electrons for *p-type* Bi_2_Te_3_ (*ξ*_p_ = 0.25, *m*_p_^*^ = 1.3 *m*_0_) are shown in figure S2 (SI).

## Additional Information

**How to cite this article**: Wang, S. *et al.* Conductivity-limiting bipolar thermal conductivity in semiconductors. *Sci. Rep.* **5**, 10136; doi: 10.1038/srep10136 (2015).

## Supplementary Material

Supplementary Information
