# Supplementary material for: Conductivity-limiting bipolar thermal conductivity in semiconductors
Source: Sci Rep. 2015 May 13;5:10136. doi: 10.1038/srep10136 (PMC4650813; doi:10.1038/srep10136)
Supplement: Supplementary Information [file srep10136-s1.docx]

**Supporting Information for**

**Conductivity-Limiting bipolar thermal conductivity in semiconductors**

Shanyu Wang^1 †^, Jiong Yang^1 †^, Trevor Toll^1^, and Jihui Yang^1^ ^‡^, Wenqing Zhang^2 ‡^, Xinfeng Tang^3 ‡^

^1^Department of Materials Science and Engineering, University of Washington, Seattle, WA 98195-2120, USA

^2^State Key Laboratory of High Performance Ceramics and Superfine Microstructure, Shanghai Institute of Ceramics, Chinese Academy of Sciences, Shanghai 200050, China

^3^State Key Laboratory of Advanced Technology for Materials Synthesis and Processing, Wuhan University of Technology, Wuhan 430070, China

1. **The effects of physical parameters on *κ*_b_**

Figure S1 shows that the *κ_b_* is more pronounced in doped semiconductors with small *E_g_* and large minority carrier effective mass. Furthermore, at a given majority carrier concentration, *κ_b_* decreases proportionally as the minority carrier mobility decreases (Figure S1(a)). The effect of the Fermi level on *κ_b_*(by changing the majority carrier concentrations), however, is not as significant (Figure S1(b)). These results also suggest, for a given material, the most effective way of altering *κ_b_* would be via modification of the minority carrier transport characteristics.

**
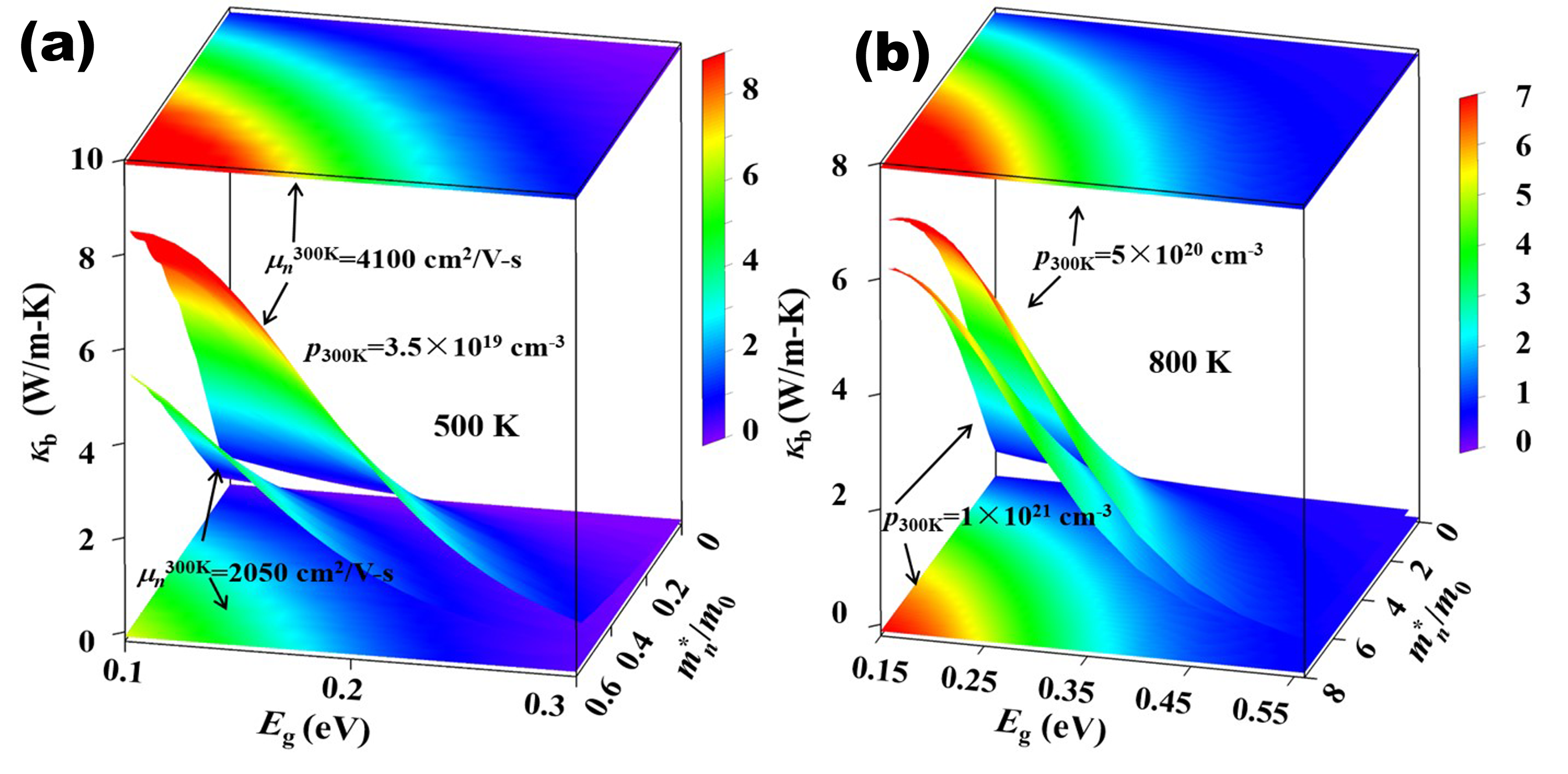
**

Figure S1. *κ_b_^Cal^* as a function of band gap (*E*_g_) and minority carrier effective mass (*m*_n_^*^) for p-type degenerate semiconductors (a) Bi_2_Te_3_ at 500 K with *p*_300K_ = 3.5×10^19^ cm^-3^ and room temperature electron mobilities *μ_n_^300K^* = 4100 and 2050 cm^2^/V-s; and (b) skutterudites at 800 K for *p*_300K_ = 5×10^20^ and 1×10^21^ cm^-3^, room temperature electron mobility *μ_n_*^300K^ = 150 cm^2^/V-s. Here *m_0_* is the free electron mass.

**2. The theoretical method of de Broglie wavelength calculations**

The de Broglie wavelengths (*λ_i_*) are defined as [^1^](#_ENREF_1)

*λ_i_ *= *h*/*m_i_*^*^*v_i_*. (1)

The subscript *i* denotes *n* (electron) or *p* (hole). For the majority carriers, density of states (DOS) effective mass *m_i_*^*^ at 300 K is estimated from the experimental Seebeck coefficient and carrier concentration, under the assumption of single parabolic band and the dominant acoustic phonon scattering ^2^. The drift velocity *v_i_* is averaged from the derivatives of bands in the vicinity of the Fermi surface based on our density functional theory (DFT) calculations. The wavelength of majority carrier is roughly energy independent. The wavelength of minority carriers, however, has a strong energy dependence, according to the semiconductor statistics. The DOS *D*(*E*) of Bi_2_Te_3_ with spin-orbit interactions was calculated via DFT, as shown in Figure S2(a).


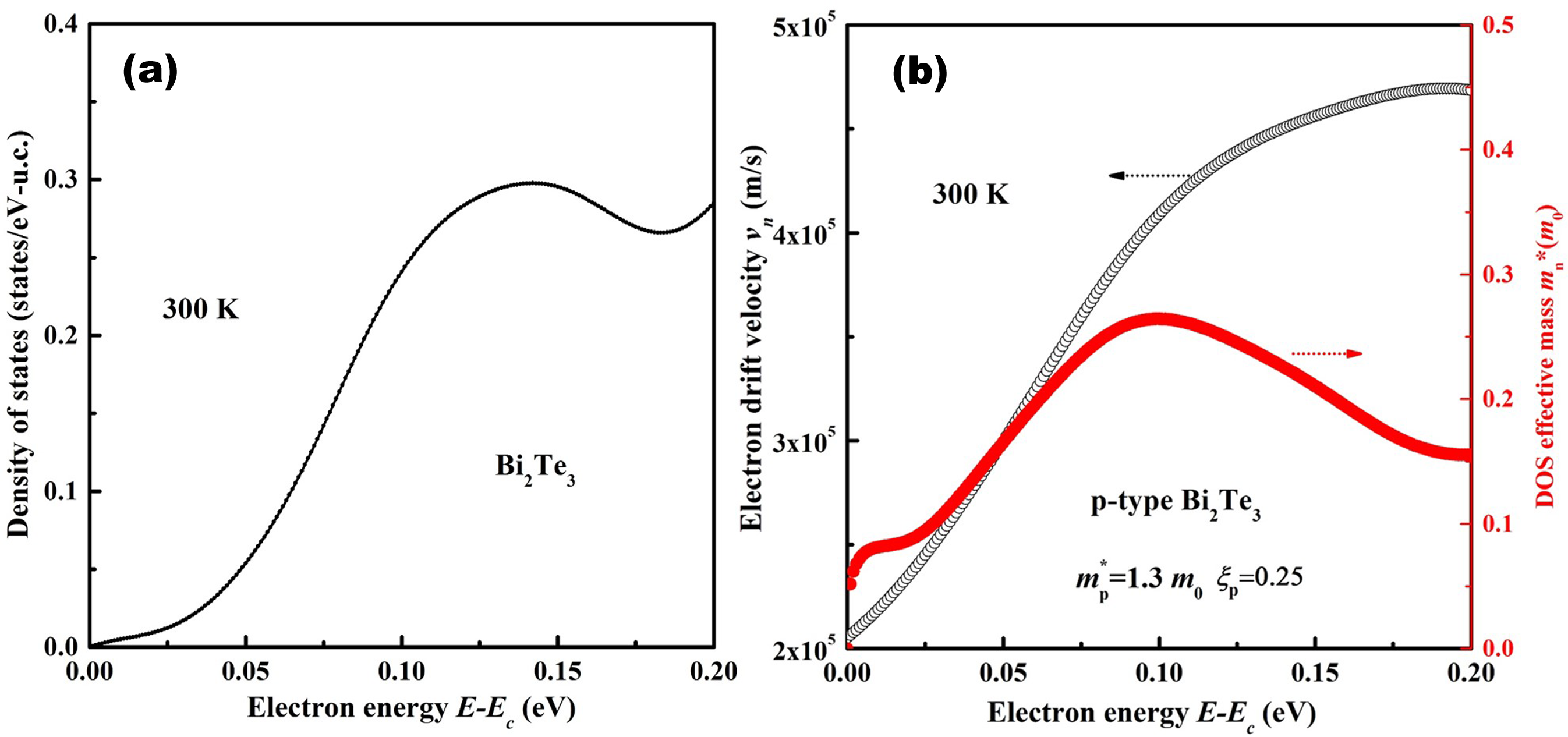

Figure S2. (a) The DOS of Bi_2_Te_3_ w.r.t. conduction band bottom (*E_c_*) ; (b) the DOS effective mass and electron drift velocity of p-type Bi_2_Te_3_ (*_p_* = 0.25, *m_p_^*^*=1.3 *m*_0_).

Based on *D*(*E*), the effective masses are calculated based on the Kane model using the following equation [^3^](#_ENREF_2)

 , (2)

where *N_v_* is the band degeneracy (*N_v_* = 6 for Bi_2_Te_3_). The calculated DOS effective masses and velocities as a function of energy are shown in Figure S2(b).

**3. Fitting parameters of hole and electron mobilities**

**Table S1.** The fitting parameters for mobility vs. carrier concentration, in Si and Ge, skutterudites (SKs), and Bi_2_Te_3_-based compounds at room temperature. The parameters of Si and Ge are taken from Ref. [4](#_ENREF_3).

| Samples | Carrier type | *i*_Ref_ (cm^-3^) | *a* | *μ*_0_ (cm^2^/V-s) | *μ*_max_(cm^2^/V-s) |
| --- | --- | --- | --- | --- | --- |
| Si | n | 1.3×10^17^ | 0.91 | 85 | 1360 |
|  | p | 5.0×10^17^ | 0.82 | 37 | 330 |
| Ge | n | 2.2×10^17^ | 0.75 | 100±20 | 3850 |
|  | p | 1.0×10^17^ | 0.56 | 40±5 | 2250 |
| SKs | n | 5.3×10^19^ | 0.90 | 0.1 | 151 |
|  | p | 6.8×10^18^ | 1.16 | 0.1 | 3060 |
| Bi_2_Te_3_-based | n | 8.7×10^17^ | 0.82 | 20+10 | 4100 |
|  | p | 2.2×10^18^ | 0.90 | 20±10 | 4600 |

**4. The parameters for bipolar thermal conductivity calculations**

**Table S2.** The parameters used for bipolar thermal conductivity calculations, here the effective mass (*m_n,p_*^*^) is determined by Seebeck coefficient and carrier concentration at 300 K. The *E*_g_s of skutterudites and Bi_2_Te_3_-based materials are determined by 2*eα*_max_*T*_max_ [^5^](#_ENREF_4), and the ***** ***^^s are the reduced Fermi levels. *E*_g_ and effective masses for Si and Ge are taken from Refs. 6-[10](#_ENREF_5). The room temperature majority carrier concentrations and mobilities are experimental data. The majority carrier concentrations in heavily doped samples are labeled in bold.

| Samples | *m_n_^*^*  (*m*_0_) | *m_p_^*^*  (*m*_0_) | *n*_300K_  (cm^-3^) | *p*_300K_  (cm^-3^) | *μ*_n_^300K^  (cm^2^/V-s) | *μ*_p_^300K^  (cm^2^/V-s) | *E*_g_  (eV) | * *^^ |
| --- | --- | --- | --- | --- | --- | --- | --- | --- |
| Yb_0.7_Fe_3_NiSb_12_ | 2.20 | 5.50 | 5.4×10^15^ | **9.0×10^20^** | 151 | 7.4 | 0.20 | 1.9 |
| YbFe_3_NiSb_12_ | 2.40 | 4.50 | 6.2×10^15^ | **4.1×10^20^** | 151 | 9.5 | 0.22 | 1.1 |
| Bi_0.5_Sb_1.5_Te_3_ (ZM) | 0.22 | 1.30 | 1.9×10^15^ | **3.5×10^19^** | 4095 | 372 | 0.18 | 0.3 |
| Bi_0.5_Sb_1.5_Te_3_ (Nano) | 0.22 | 1.30 | 1.1×10^15^ | **3.3×10^19^** | 1115 | 390 | 0.20 | 0.2 |
| Bi_2_Te_2.7_Se_0.3_ (ZM) | 1.20 | 0.11 | **3.2×10^19^** | 1.3×10^15^ | 243 | 4614 | 0.16 | 0.3 |
| Si single crystal [^11^](#_ENREF_10) | 0.32 | 0.50 | 2.3×10^9^ | 2.3×10^9^ | 1445 | 367 | $1.17-\frac{4.73\times{10}^{-4}T^{2}}{T+636}$ | -21.4 |
| Ge single crystal [^11^](#_ENREF_10) | 0.12 | 0.30 | 2.5×10^12^ | 2.5×10^12^ | 3969 | 2289 | $0.785-\frac{4.77\times{10}^{-4}T^{2}}{T+235}$ | -12.9 |

**Table S3.** The parameters used for calculating the bipolar thermal conductivity of Ba_x_Fe_2_Co_2_Sb_12_ and Ba_y_FeCo_3_Sb_12_. *E*_g_s are determined by 2*eα*_max_*T*_max_ [^5^](#_ENREF_4), and ***** ***^^s are the reduced Fermi levels. The room temperature majority carrier concentrations and mobilities are experimental data. The majority carrier concentrations in heavily doped samples are labeled in bold.

| Samples | *m_n_^*^*  (*m*_0_) | *m_p_^*^*  (*m*_0_) | *n*_300K_  (cm^-3^) | *n*_800K_  (cm^-3^) | *p*_300K_  (cm^-3^) | *μ*_n_^300K^  (cm^2^/V-s) | *μ*_p_^300K^  (cm^2^/V-s) | *E*_g_  (eV) | * *^^ |
| --- | --- | --- | --- | --- | --- | --- | --- | --- | --- |
| Ba_0.85_Fe_2_Co_2_Sb_12_ | 2.1 | 1.5 | 8.7×10^13^ | 1.9×10^18^ | **2.3×10^20^** | 151 | 22 | 0.27 | 3.3 |
| Ba_0.95_Fe_2_Co_2_Sb_12_ | 2.1 | 1.5 | 2.7×10^14^ | 3.0×10^18^ | **1.4×10^20^** | 151 | 27 | 0.27 | 2.1 |
| BaFe_2_Co_2_Sb_12_ | 2.1 | 1.5 | 7.9×10^14^ | 4.5×10^18^ | **7.5×10^19^** | 151 | 41 | 0.27 | 1.0 |
| Ba_0.35_FeCo_3_Sb_12_ | 1.4 | 1.5 | 1.3×10^14^ | 1.6×10^18^ | **2.1×10^20^** | 151 | 25 | 0.25 | 3.1 |
| Ba_0.45_FeCo_3_Sb_12_ | 1.3 | 1.5 | 3.2×10^14^ | 2.0×10^18^ | **1.3×10^20^** | 151 | 29 | 0.25 | 2.0 |
| Ba_0.5_FeCo_3_Sb_12_ | 1.2 | 1.5 | 7.5×10^14^ | 2.6×10^18^ | **7.4×10^19^** | 151 | 41 | 0.25 | 1.0 |

**References**

1. Blakemore, J. S. *Semiconductor Statistics*. (Courier Dover Publications, 2002).
2. Wang, S. et al. The realization of a high thermoelectric figure of merit in Ge-substituted β-Zn_4_Sb_3_ through band structure modification. *J. Mater. Chem.* 22, 13977-13985 (2012).
3. Huang, B.-L. & Kaviany, M. Ab initio and molecular dynamics predictions for electron and phonon transport in bismuth telluride. *Phys. Rev. B* **77**, 125209 (2008).
4. Slack, G. A. & Hussain, M. A. The maximum possible conversion efficiency of silicon‐germanium thermoelectric generators. *J. Appl. Phys.* **70**, 2694-2718 (1991).
5. Goldsmid, H. & Sharp, J. Estimation of the thermal band gap of a semiconductor from Seebeck measurements. *J. Electron. Mater.* **28**, 869-872 (1999).
6. Lautenschlager, P., Allen, P. & Cardona, M. Temperature dependence of band gaps in Si and Ge. *Phys. Rev. B* **31**, 2163 (1985).
7. Thurmond, C. The standard thermodynamic functions for the formation of electrons and holes in Ge, Si, GaAs, and GaP. *J. Electrochem Soc.* **122**, 1133-1141 (1975).
8. Spitzer, W. & Fan, H. Infrared absorption in n-type silicon. *Phys. Rev.* **108**, 268 (1957).
9. Fischetti, M. V. & Laux, S. E. Band structure, deformation potentials, and carrier mobility in strained Si, Ge, and SiGe alloys. *J. Appl. Phys.* **80**, 2234-2252 (1996).
10. Green, M. A. Intrinsic concentration, effective densities of states, and effective mass in silicon. *J. Appl. Phys.* **67**, 2944-2954 (1990).
11. Glassbrenner, C. & Slack, G. A. Thermal conductivity of silicon and germanium from 3 K to the melting point. *Phys. Rev.* **134**, A1058 (1964).
